# Animal Models of Bone Marrow Lesions in Osteoarthritis

**DOI:** 10.1002/jbm4.10609

**Published:** 2022-02-15

**Authors:** Andrew Bowen, David Shamritsky, Josue Santana, Ian Porter, Erica Feldman, Sarah L Pownder, Matthew F Koff, Kei Hayashi, Christopher J Hernandez

**Affiliations:** ^1^ Sibley School of Mechanical and Aerospace Engineering Cornell University Ithaca NY USA; ^2^ Meinig School of Biomedical Engineering Cornell University Ithaca NY USA; ^3^ College of Veterinary Medicine Cornell University Ithaca NY USA; ^4^ Hospital for Special Surgery New York NY USA

**Keywords:** BONE, BONE MARROW LESION, JOINT, MICRODAMAGE, OSTEOARTHRITIS

## Abstract

Bone marrow lesions are abnormalities in magnetic resonance images that have been associated with joint pain and osteoarthritis in clinical studies. Increases in the volume of bone marrow lesions have been associated with progression of joint degeneration, leading to the suggestion that bone marrow lesions may be an early indicator of—or even a contributor to—cartilage loss preceding irreversible damage to the joint. Despite evidence that bone marrow lesions play a role in osteoarthritis pathology, very little is known about the natural history of bone marrow lesions and their contribution to joint degeneration. As a result, there are limited data regarding the cell activity within a bone marrow lesion and any associated bone‐cartilage cross‐talk. Animal models provide the best approach for understanding bone marrow lesions at their early, reversible stages. Here, we review the few animal studies of bone marrow lesions. An ideal animal model of a bone marrow lesion occurs in joints large enough to accurately measure bone marrow lesion volume. Additionally, the ideal animal model would facilitate the study of bone‐cartilage cross‐talk by generating the bone marrow lesion immediately adjacent to subchondral bone and would do so without causing direct damage to neighboring soft tissues to isolate the effects of the bone marrow lesion on cartilage loss. Early reports demonstrate the feasibility of such an animal model. Given the irreversible nature of osteoarthritic changes in the joint, factors such as bone marrow lesions that are present early in disease pathogenesis remain an enticing target for new therapeutic approaches. © 2022 The Authors. *JBMR Plus* published by Wiley Periodicals LLC on behalf of American Society for Bone and Mineral Research.

## 1 Introduction

**Fig. 1:**
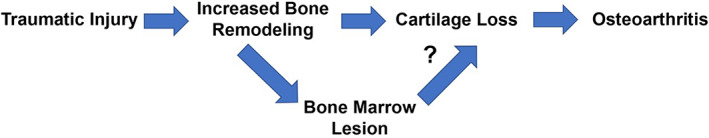
Hypothesized association of bone marrow lesions with osteoarthritis after trauma. Derived from Alliston and colleagues.^(^ ^2^ ^)^

Bone marrow lesions (BMLs) are regions of high‐intensity signal displayed using magnetic resonance imaging (MRI) fat suppressed techniques such as T2 fat suppression, short tau inversion recovery (STIR), or Dixon methods.^(^ ^1^ ^)^ The presence and/or progression of BMLs has been associated with joint pain and/or the development of osteoarthritis (OA). Evidence suggesting that the appearance of a BML precedes substantial joint degeneration elicits the possibility that BMLs may be a noninvasive indicator of subsequent joint degeneration.^(^ ^2^ ^)^ Additionally, recent findings regarding bone‐cartilage cross‐talk suggest the possibility that processes within the BML can contribute to cartilage loss (Fig. 1).

Historically, BMLs have been referred to as “bone marrow edema” or “bone bruise,” but the term “bone marrow lesion” is preferred because most cases lack other traits of edema.^(^ ^1^ ^)^ Similar abnormalities on MRI are observed in the vertebral endplates and are referred to as “type I Modic changes.”^(3^ ^)^ Type I Modic changes are associated with pain and share many of the histopathologic characteristics found in BMLs in the joints of the appendicular skeleton^(^ ^3^ ^)^ (see below). The current discussion will focus on BMLs of the knee, although we recognize that much of the discussion may also be relevant to understanding Modic changes in the spine.^(^ ^3^ ^)^

Roemer and colleagues developed classifications of BMLs in the knee based on clinical correlates rather than the underlying physiology, as the process of BML pathogenesis was (and still is) poorly understood.^(^ ^1^ ^)^ BMLs are first classified as traumatic or nontraumatic. Traumatic BMLs are associated with clear subchondral fracture, impaction, or stress fracture, and/or with repetitive overuse. The well‐recognized “bone bruise” or bone contusion observed in trauma is a common form of traumatic BML. Traumatic BMLs often resolve within months after an injury if a proper rehabilitation regimen is prescribed and followed.^(^ ^1^, ^4^ ^)^ Nontraumatic BMLs include those associated with subchondral necrosis as well as those associated with osteoarthritis. Nontraumatic BMLs, especially those associated with osteoarthritis, can be present for months to years and may never fully resolve.^(^ ^1^ ^)^

Although BMLs are commonly observed clinically, very little is known about their etiology and pathogenesis, the cellular processes within the BML, and under what conditions the BMLs are associated with cartilage degeneration. Here, we review clinical observations of BMLs (both invasive and noninvasive) and their association with progression of osteoarthritis, and we discuss approaches to developing animal models to study the pathogenesis of BMLs and their relationship to joint degeneration.

## 2 Clinical Studies Linking BMLs to Osteoarthritis

The study of BMLs in the context of knee OA has primarily focused on (i) correlations between BML volume and perceived knee pain; (ii) the predictive capacity for deteriorating joint health; and (iii) whether receding BMLs are associated with improved patient symptoms.

Several studies have considered the degree to which BML volume is useful for predicting the onset and severity of cartilage disease and osteoarthritis. Davies‐Tuck and colleagues^(^ ^5^ ^)^ analyzed the knees of an asymptomatic, non‐OA, and injury‐free cohort (*n* = 271) and found the incidence of a medial BML to be associated with the progression of medial tibiofemoral cartilage loss. The presence of a BML was associated with worsening of overall joint health over a 1‐year period (odds ratio [OR] = 2.63, 95% confidence interval [CI] 0.93, 7.44). Additionally, cartilage loss (measured as thickness lost) on the tibia over a 1‐year period was greater in individuals in which a BML was present in more than three consecutive MRI slices. In contrast, individuals in which the BML resolved over the study period showed less annual volume loss of cartilage.^(^ ^5^ ^)^

The effect of baseline BML on subsequent progression of a cartilage defect was analyzed in a random sample of adults aged 51 to 81 years (*n* = 405).^(^ ^6^ ^)^ The presence and volume of a local BML predicted the magnitude of cartilage defect progression as well as cartilage volume loss. Absolute volume loss of cartilage is larger on the tibial plateau compared with the femoral condyles, but the presence of a BML is associated with a twofold increase in cartilage loss on both joint surfaces. Additionally, the presence of an initial cartilage defect operates in concert with the presence of a BML to further exacerbate cartilage loss, suggesting that the two induce cartilage loss through additive yet distinct pathways.^(^ ^6^ ^)^

Studies using the Osteoarthritis Initiative (OAI) database have clearly associated BMLs with accelerated knee osteoarthritis (AKOA). AKOA is defined as progression from no radiographic abnormality to advanced osteoarthritis in less than 4 years.^(^ ^7^ ^)^ The relatively fast development of AKOA makes it more likely to be observed in longitudinal studies and therefore likely to be observed in association with an early‐stage symptom like a BML. This analysis shows that individuals with a BML identified a year before the diagnosis of OA were more likely to experience AKOA. Furthermore, BML volume was greater in individuals who went on to develop AKOA compared with individuals with typical osteoarthritis or no osteoarthritis.^(^ ^8^ ^)^

Bone marrow lesions are also common in individuals who require knee arthroplasty. Patients with an MRI BML signal are nine times more likely to undergo a total knee arthroplasty within the next 3 years, even after accounting for age.^(^ ^9^ ^)^ Just before arthroplasty, individuals with BMLs in the tibial plateau showed lower cartilage volume and higher OARSI scores than non‐BML controls.^(^ ^10^ ^)^ Within a whole joint, BML presence in the femoral condyles and tibial plateau is predictive of the magnitude of cartilage loss (both thickness and volume) and worsening overall joint health. Although differences exist between the tibia and femoral cartilage surfaces and between medial and lateral compartments, the trends are well conserved. These two large cohort studies are some of the only studies that examine BMLs in a prospective manner, ie, obtaining longitudinal images before the explicit onset of OA symptoms, and as such are a valuable resource for developing a novel model of BMLs.

## 3 Histopathological Changes in Bone Within a Bone Marrow Lesion

The pathogenesis of BMLs is not yet clear. The high‐intensity signal characteristic of BMLs in MR images indicates a region with increased water content; however, histological analyses indicate a very limited presence of edema (4% of samples).^(^ ^1^, ^11^ ^)^ Histological analysis of tissue collected from BMLs at the time of arthroplasty indicates increased bone volume fraction, microscopic damage within the bone tissue, fibrosis, increased vascularization, and reduced bone mineral content.^(^ ^10^, ^11^, ^12^, ^13^, ^14^, ^15^, ^16^, ^17^, ^18^, ^19^ ^)^ Some reports indicate necrotic bone marrow and subchondral trabeculae, which are thicker and more mineralized than their counterparts not within lesions.^(^ ^12^, ^20^ ^)^ Others have reported reduced mineralization of subchondral bone within a BML consistent with increased rates of bone remodeling.^(^ ^21^, ^22^ ^)^ The density of vascular channels in subchondral bone adjacent to calcified cartilage is greater within a BML.^(^ ^20^ ^)^ Additionally, transcriptome analysis reveals that BMLs highly express genetic signatures representing neuronal development, pain, angiogenesis, and pathways involved in cytokine signaling.^(^ ^23^ ^)^

Multiple studies have shown that bone remodeling is increased in regions of BMLs.^(^ ^10^, ^21^, ^24^ ^)^ The increase in bone remodeling within a BML led to the idea that suppression of bone remodeling using bisphosphonates might stop or reverse the growth of BMLs within the joint.^(^ ^20^ ^)^ However, clinical findings have been mixed; some have indicated that bisphosphonates can reduce the volume of the BML,^(25^ ^)^ whereas others did not observe a significant effect on BML size,^(^ ^26^ ^)^ suggesting that expansion of an established BML is not driven by remodeling alone. The clinical study focused on individuals with late‐stage osteoarthritis requiring arthroplasty, however, and it remains possible that antiresorptive agents can interrupt BMLs and associated pain earlier in joint pathogenesis. Some bisphosphonates, such as zoledronic acid, have demonstrated anti‐angiogenic properties that suppress vascularization.^(^ ^27^ ^)^ The increase in bone remodeling within a BML may also have an effect on the material properties of subchondral bone tissue: The tissue bone mineral density distribution is skewed toward lower mineralization compared with regions outside of a BML.^(^ ^22^ ^)^ This finding is consistent with the presence of more‐recently formed bone tissue with incomplete secondary mineralization.

Shabestari and colleagues examined subchondral bone from hip joints with bone marrow lesions collected at the time of arthroplasty from individuals with OA. The subchondral bone showed an increased mineralizing surface as well as mineral apposition consistent with increased bone remodeling.^(^ ^28^ ^)^ This finding indicates that trabecular bone remodeling is increased within a BML. Additionally, the region of the BML was found to have more woven bone, suggesting the presence of repair mechanisms after mechanical damage.^(^ ^28^ ^)^ Despite indications of increased bone remodeling, there were no indications of altered bone volume fraction within the BMLs.

Although the studies mentioned here have provided insights into the histopathology of BMLs, they have all been limited in scope as the tissues were collected at time of surgery. As a result, the observations are characteristic of BMLs in late‐stage osteoarthritis, which may not have a profile representative of BMLs during early‐stage cartilage degeneration. Histopathology at an earlier stage—when a BML is present but joint degeneration has not yet been established—would provide more useful information regarding the utility of the BML as an imaging biomarker or a therapeutic target. Patient biopsies of a BML at such an early stage of development are not possible, so animal models may provide a means to understand the etiology and pathogenesis of BMLs and their potential contribution to osteoarthritis.

## 4 Bone Marrow Lesions in Animal Models of Posttraumatic Osteoarthritis

**Fig. 2:**
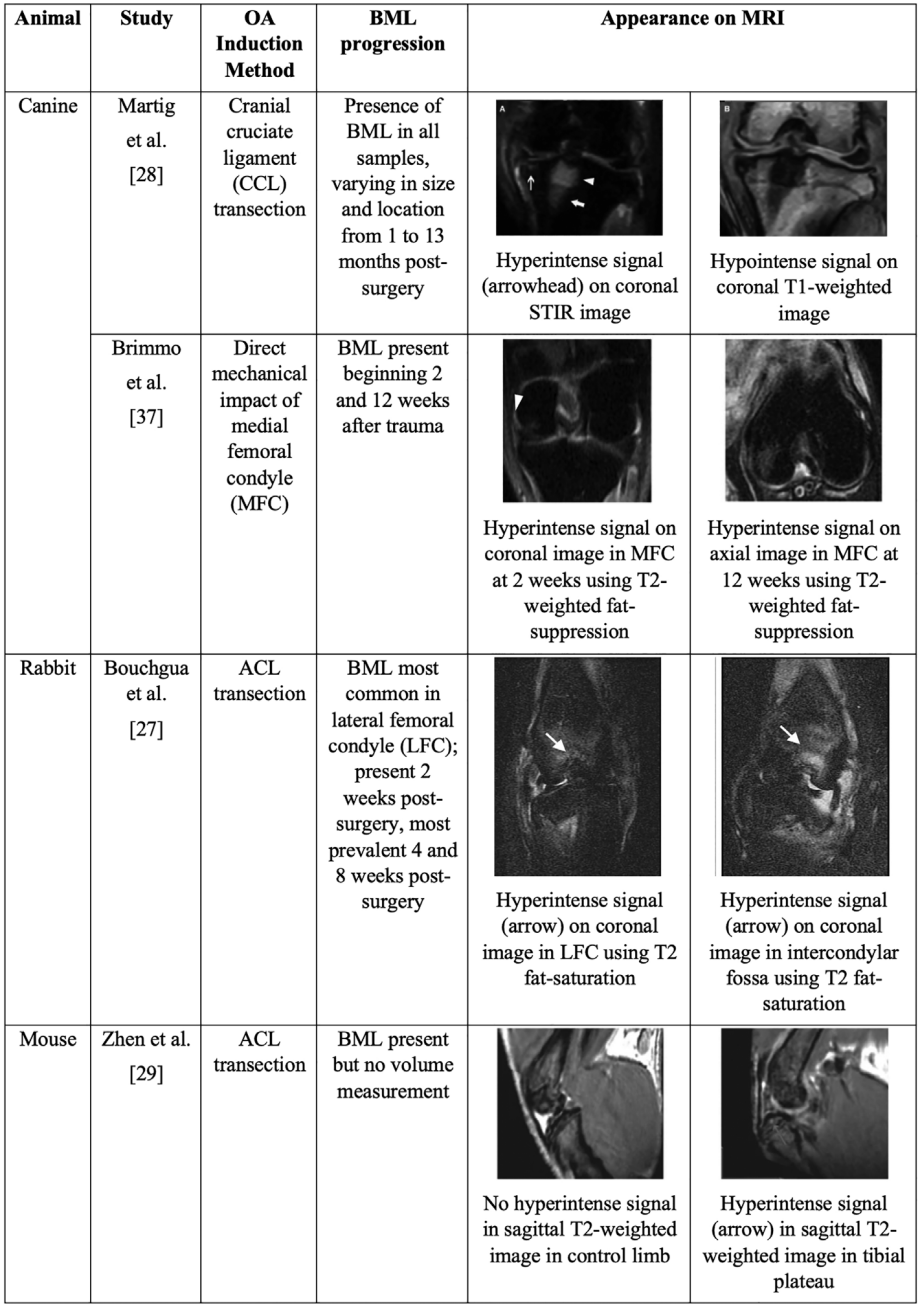
Comparison of findings from previous studies measuring bone marrow lesions via MRI in animal models of traumatic surgical induction of osteoarthritis. Reprinted by permission from John Wiley and Sons: Veterinary Radiology & Ultrasound.^(^ ^31^ ^)^ Reprinted by permission from Georg Thieme Verlag KG: Journal of Knee Surgery.^(^ ^40^ ^)^ Reprinted by permission from Elsevier: Osteoarthritis and Cartilage.^(^ ^30^ ^)^ Reprinted by permission from Springer Nature: Nature Medicine.^(^ ^32^ ^)^

Animal models of osteoarthritis primarily simulate posttraumatic osteoarthritis through injury or surgical trauma. Here, we summarize studies in which MRI capable of detecting bone marrow lesions was used in the context of an animal model of osteoarthritis (Fig. 2).

Surgical transection of the anterior cruciate ligament (ACL) is a well‐established approach for initiating cartilage loss and joint degeneration in animals. ACL transection has been implemented in animals ranging in size from mice to dogs and repeatably leads to cartilage loss within weeks of surgery.^(^ ^29^ ^)^ In rabbits, the presence of a BML was more common in animals that had ACL transection surgery (8/12 joints) than sham limbs (1/12 joints) or nonsurgical controls (3/12 joints). Furthermore, BMLs were detected as soon as 2 weeks after ACL transection but were more common at 4 and 8 weeks post‐surgery.^(^ ^30^ ^)^ In a canine model, cranial cruciate ligament (CCL) transection yielded BMLs that were first detectable 1 month post‐surgery and remained present on MRI for at least 13 months.^(^ ^31^ ^)^ The BMLs within the canine joints varied in size and volume without a recognizable trend over time. These two studies demonstrate an association between BMLs and cartilage loss but not causality, and they provide little information about mechanisms linking BMLs to joint degeneration.

Bone marrow lesions have also been observed in mice after ACL transection.^(^ ^32^ ^)^ The BMLs in mice were small in volume and associated with local increases in TGF‐beta within the subchondral bone as well as increased bone resorption, increased presence of remodeling cavities, and increased angiogenesis at 30 days post‐surgery. Furthermore, inhibiting TGF‐beta post‐ACL transection resulted in smaller BMLs, reduced subchondral bone remodeling, and slower cartilage degradation.^(^ ^32^ ^)^ In a follow‐up study by the same group, increased osteoclast activity within the subchondral bone soon after ACL transection was associated with increased pain sensation through osteoclast secretion of Netrin‐1.^(^ ^33^, ^34^ ^)^ More recently, changes in subchondral bone structure associated with ACL transection have been shown to modify the stress distributions in neighboring articular cartilage,^(^ ^35^ ^)^ suggesting that modifications to the underlying subchondral bone may cause direct changes in cartilage stress and strain (consistent with prior finite element modeling^(^ ^36^ ^)^). This set of findings is exciting in that it suggests mechanisms that can link bone cell activity (such as osteoclast activity) to both joint pain and mechanical degradation of the articular cartilage. However, a limitation of this line of work with regard to BMLs is that only one of these studies included MR imaging capable of detecting a BML,^(^ ^32^ ^)^ and the volume of the BMLs were not directly measured. Other MR imaging approaches may be needed to measure the volume of a BML in the mouse joint.^(^ ^37^ ^)^ Follow‐up studies are needed to confirm the presence of a BML in this mouse model and, if present, whether it precedes joint degeneration or is simply co‐occurring. Another limitation of this line of investigation is the use of ACL transection, which causes severe changes in joint stability to initiate osteoarthritis (the ACL transection model is classified as “traumatic” in the literature^(^ ^38^, ^39^ ^)^). It is unclear if the same mechanisms identified under this severe traumatic disruption of joint biomechanics are also active in less‐severe situations such as nontraumatic OA‐associated BMLs.

Direct mechanical overload of the cartilage and joint has also been used to induce cartilage degeneration and bone marrow lesions. Brimmo and colleagues developed a canine model in which a singular impact at the center of the cartilage surface on the medial femoral condyle generates the initial insult.^(^ ^40^ ^)^ Loading in this focal manner causes cartilage defects as well as damage in subchondral bone. At 2 weeks after loading, BMLs are present, and at 12 weeks after injury, the grade of the BML (radiologic score) was greater in animals that received a larger magnitude of applied load. The model is promising, although it would benefit from additional characterization such as incorporating a longitudinal evaluation. It is important to recognize that each of the studies highlighted in this review use exclusively skeletally mature male animals in their surgical models of OA. The OARSI histopathology initiative notes that although both male and female rabbits are used in the literature, the effects of fluctuating hormones are unclear, and male animals may be preferable for initial studies.^(^ ^41^ ^)^

Despite only a subset of animal studies of osteoarthritis using MRI capable of detecting BMLs, most have indicated the presence of a BML in situations with cartilage loss. In the animal studies discussed, BMLs are only observed in situations in which there is a direct mechanical overload and/or damage to the overlying cartilage, and/or changes in joint kinematics (as observed in late‐stage osteoarthritis in humans and ACL transection in animals). The close association between BMLs and cartilage damage makes it difficult to understand mechanisms of bone‐cartilage cross‐talk in this situation. To truly understand the role of BMLs in the pathogenesis of osteoarthritis, it is necessary to examine BMLs before, or in the absence of, mechanical overload to the overlying cartilage.

**Fig. 3:**
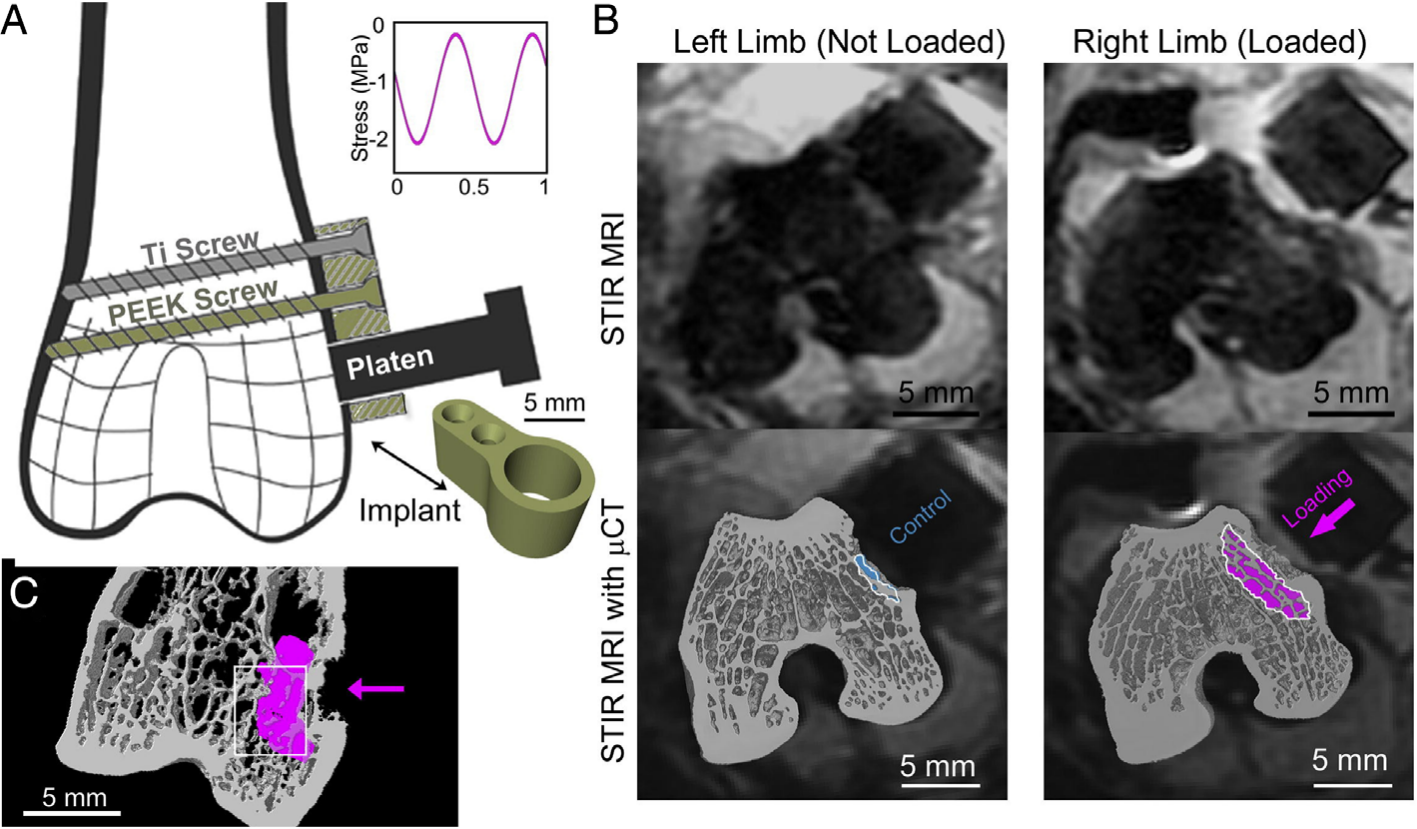
A rabbit model of a bone marrow lesion in epiphyseal bone. (*A*) Placement of the implant and alignment of loading platen. (*B*) MR images at 2 weeks after surgery/loading: Raw image (top) with overlay from micro–computed tomography with the bone marrow lesion colored (bottom). (*C*) Micro–computed tomography image with overlay of the bone marrow lesion (pink). From Matheny and colleagues,^(^ ^24^ ^)^ used with permission.

To address this challenge, we developed an animal model in which a bone marrow lesion observable with MRI could be generated within bone without concomitant cartilage damage^(^ ^24^ ^)^ (Fig. 3). In its initial implementation, the experimental model involved a surgical approach in which a polyether ethyl ketone (PEEK) implant is secured to the distolateral femur of a skeletally mature (7‐month‐old) adult intact male rabbit. A routing tool is then applied through a hole in the implant to remove a region of the cortex below the implant (1 mm depth). A flat metallic plunger connected to a materials testing device is then used to apply a cyclic mechanical load directly to the cancellous bone revealed by removal of the cortex. After cyclic loading is completed, a cap is secured within the implant as a means of sealing the hole in the cortex. At 2 weeks after surgery/loading, a BML was present in the underlying cancellous bone, and it was significantly larger than the small BML‐like signal present in sham surgery non‐loaded limbs. Additionally, the presence of the BML is associated with microscopic tissue damage in the underlying cancellous bone and increased bone resorption activity (as indicated by increased eroded surface) at 2 weeks after surgery/loading. Lastly, the volume of the BML was directly correlated with the amount of microscopic tissue damage in the underlying cancellous bone. This finding represents the first animal model of a BML that allows direct examination of both the microscopic bone tissue damage and the associated bone remodeling responses within a BML. However, the location of the BML was distant from the articular cartilage, thereby limiting the ability of the model to assess the association of the BML with cartilage damage. To better understand the relationship between BMLs and cartilage loss, a new animal model is needed.

## 5 Animal Models of Bone Marrow Lesions

Here we review the challenges in developing an animal model of a BML. Although there are non‐mechanical causes of BMLs (inflammation, tumors, avascular necrosis, etc.), the vast majority of traumatic and non‐traumatic BMLs (including “osteoarthritis associated” BMLs) include increases in mechanical load within the subchondral bone. As a result, mechanical stimulation of the bone is the most attractive means for generating a BML like those most often found in human patients. There are four factors that must be considered when establishing an animal model of a BML: (i) the animal species to be used; (ii) the location of the applied mechanical load; (iii) the loading waveform (magnitude, frequency, number of cycles of loading); and (iv) the minimization of surgical trauma involved when generating the BML.

First, the laboratory animal selected for the work should have a sufficient volume of subchondral cancellous bone so that the volume of the resulting BML can be reliably measured. Additionally, the study should be performed in skeletally mature animals with fused growth plates to prevent potential influences of endochondral ossification processes on the natural development of the BML. These criteria limit the utility of mice and rats for the study of bone marrow lesions because the size of rodent bones is small compared with the voxel sizes in most MR systems. Additionally, the growth plates of long bones in mice and rats do not fuse until very late in life (if at all), and an active neighboring growth plate may influence processes within the BML. Larger animals including dogs and rabbits may be preferred for the study of BMLs (consistent also with prior work, see above). Conceivably, large animals such as sheep and goats could also be used for this purpose, but the added expense of housing and caring for these animals presents a challenge.

Second, the location of the applied load is extremely important from both surgical and anatomical perspectives. To study interactions between the BML and neighboring cartilage, the BML must be generated immediately adjacent to the articular cartilage. Although direct application of mechanical loads through the cartilage has been shown to generate a BML,^(^ ^40^ ^)^ the approach also causes direct mechanical damage to the cartilage. This makes it difficult to separate the effects of the BML on neighboring cartilage from the effect of the mechanical load. A secondary consideration is the load distribution across the joint during normal locomotion of the animal, as the response to the BML is likely to be greater in regions experiencing larger loads during ambulation. Lastly, applying the loads in a way that also allows subsequent application of other models of osteoarthritis (ACL transection, destabilization of the medial meniscus, partial meniscectomy) is attractive because it permits studies to examine the effects of a BML in concert with different means of initiating OA. Such a design could be valuable for investigating whether the coexistence of a BML has any effect on the rate of development and/or severity of osteoarthritis resulting from other known induction methods.

Once the anatomical location is determined, a specific load magnitude or range of load magnitudes to generate the BML must be determined. Available histological data associate the presence of a BML with microscopic tissue damage in cancellous bone, including microscopic cracks, diffuse damage, and trabecular microfracture.^(^ ^11^, ^15^, ^20^, ^24^, ^42^ ^)^ These findings suggest that the presence of microscopic tissue damage in bone is required to replicate the majority of BMLs observed clinically. In humans, traumatic BMLs are often caused by impact loads such as a fall or associated with severe soft tissue damage (ie, ACL tears). Although impact loading could be used to generate a BML in an animal, it is difficult to implement such a load without causing a fracture in the cortical shell, which causes additional inflammatory responses that may confound the study of the BML itself. Alternatively, microdamage in cancellous bone is readily generated through the application of cyclic loading.^(^ ^43^, ^44^ ^)^ Generating microscopic tissue damage in cancellous bone requires a balance between the maximum load magnitude and the number of loading cycles. Too large of a maximum load magnitude will cause overt fracture and local crushing, while too small of a maximum load will only generate microdamage after excessively long periods of loading—too long for application to a live animal.^(^ ^24^, ^45^ ^)^ Hence, careful calibration of the magnitude and number of cycles of applied loading is required to repeatably generate tissue microdamage in cancellous bone. We have demonstrated such an approach when loads are applied directly to underlying cancellous bone,^(^ ^24^ ^)^ and it is also possible to generate microscopic tissue damage in cancellous bone without removal of the cortex.^(^ ^45^ ^)^

Lastly, the ideal animal model of a BML should involve minimal surgical trauma because surgical trauma alone can cause a BML.^(^ ^1^, ^24^ ^)^ If the BML generated by the surgical trauma is too large and/or persists for too long, it could overwhelm the desired bone marrow lesion to be generated by applied loading. This makes it difficult to separate responses associated with surgical trauma from those associated with the load‐induced BML.

## 6 An Example of an Animal Model of a Bone Marrow Lesion

Here, we present preliminary data describing the early steps in developing an animal model for generating a BML in subchondral bone. As in our prior work, the rabbit is selected as the animal of choice because it has a sufficiently large subchondral bone volume for MR imaging, and the growth plates of the distal femur are fused at a reasonable age (~6 months).^(^ ^24^ ^)^ This implementation differs from our prior work in three ways: (i) the location of the applied load was moved from the subchondral epiphysis to a region immediately adjacent to (but not touching) articular cartilage in order to generate the BML adjacent to subchondral bone; (ii) the location of applied load was moved to the medial (rather than lateral) side of the joint to allow subsequent use of other means of stimulating cartilage loss (ie, destabilization of the medial meniscus); and (iii) surgical trauma of the model was reduced by applying the loads without removing the cortex. These modifications in the model made it possible to remove the fixture used to align the loading system at the end of the procedure, reducing postoperative discomfort to the animals and improving the quality of the MR images (which may have susceptibility artifact present due to an implanted device). Live animal work was performed after approval by the local institutional animal care and use committee (IACUC).

The new anatomical location for the BML‐inducing mechanical loads required a preliminary study to determine an appropriate load magnitude and number of cycles sufficient for inducing a BML. Given the strong association between microscopic tissue damage in bone and BML size,^(^ ^24^ ^)^ an appropriate loading waveform should also generate microscopic tissue damage in underlying cancellous bone. Our prior work found that applying mechanical loads to cancellous bone from zero to a maximum load corresponding to 2 MPa at 2 Hz for 10,000 cycles reliably generated microdamage in cancellous bone.^(^ ^24^ ^)^ The same frequency and number of cycles was selected to achieve a period of loading (83 minutes) that was acceptable as determined by institutional veterinary consults while keeping the load frequency close to physiologic loading frequency (~1 Hz).

**Fig. 4:**
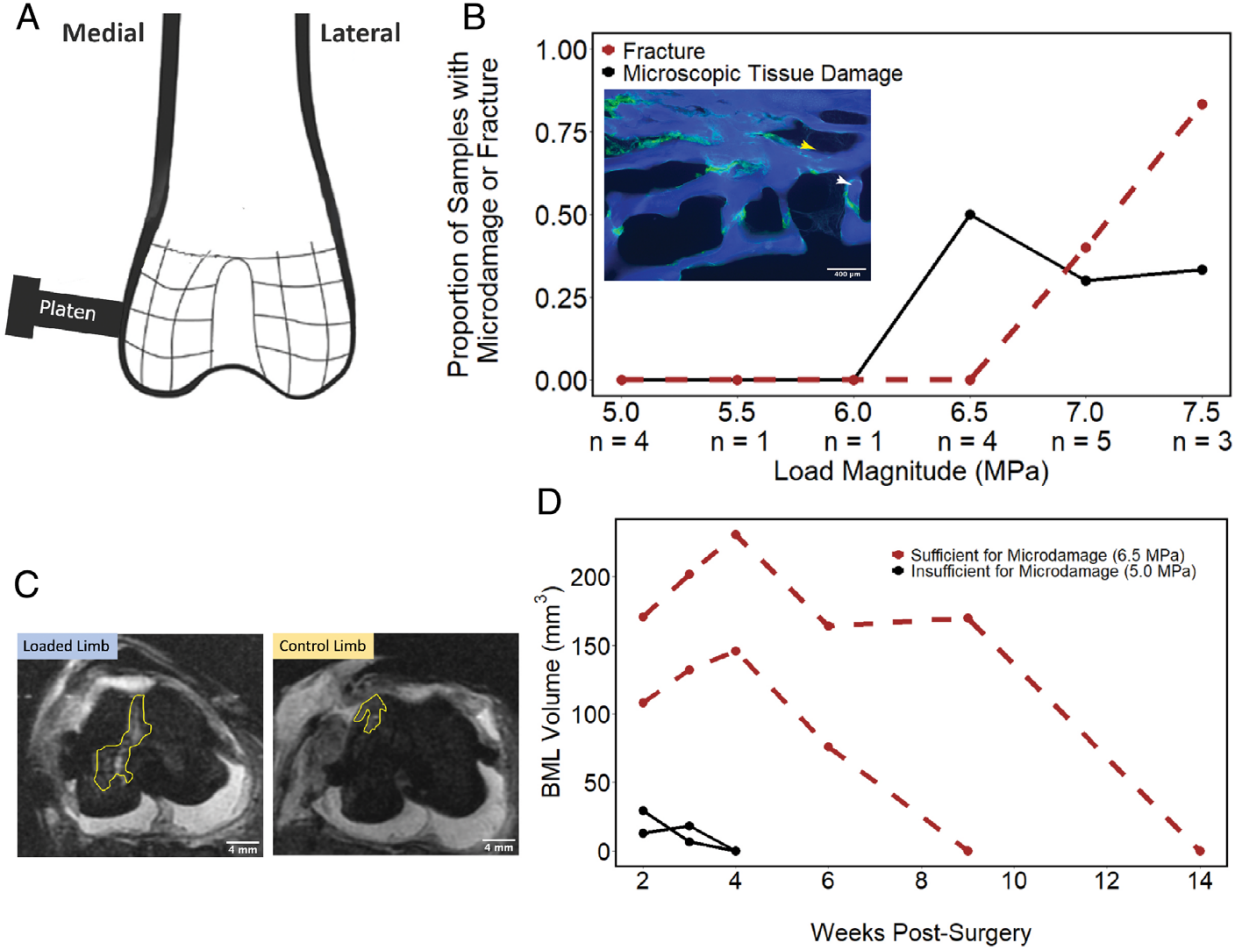
(*A*) The position of the mechanical load used to induce the bone marrow lesion on the distal medial femur is shown. (*B*) The relationship between maximum load magnitude and the presence of microscopic tissue damage or overt failure of the cortical shell. Number of joints at each load magnitude is shown in the bottom row. (Inset) Microscopic tissue damage (green) in underlying cancellous bone (blue) caused by the selected loading waveform is shown. Yellow arrowhead = microdamage; white arrowhead = microfracture. (*C*) STIR MR images with bone marrow lesions are shown. (*D*) The volume of bone marrow lesions over time is shown for mechanical loading insufficient to generate microscopic tissue damage and mechanical loading sufficient to generate microscopic tissue damage. Each line represents a single limb studied longitudinally (*n* = 2 for each type).

After IACUC approval, a preliminary study was performed using male New Zealand white rabbits, 6 to 7 months of age (Charles River Laboratories, Wilmington, MA, USA). Animals were euthanized, and cadavers were subjected to bilateral surgical insertion of an aluminum fixture on the distal femur. The fixtures were secured to the bones using 1.5‐mm‐diameter, 20‐mm‐length, non‐self‐tapping, veterinary cortex screws (VS101.020, DePuy Synthes Vet, West Chester, PA, USA). The hindlimb was then aligned beneath a vertically mounted servoelectric materials testing device (Testbench, Bose Electroforce, Eden Prairie, MN, USA). A 6‐mm‐diameter cylindrical loading platen was then inserted through the alignment hole in the fixture, and cyclic loading (zero to compression) was applied. Each limb received cyclic loading with a maximum load magnitude ranging from 5.0 to 7.5 MPa (supraphysiologic load; one load magnitude per limb, *n* = 1–5 limbs per load magnitude). Two additional limbs were positioned within the materials testing device, but no load was applied (sham loading). After loading, the fixtures were removed, and the bones were dissected. Distal femoral heads were isolated, marrow was removed, and samples were bulk stained in calcein for 2 hours under vacuum to stain microscopic tissue damage. Distal femoral heads were then embedded undecalcified in polymethyl methacrylate and sectioned for analysis (as done previously).^(^ ^46^ ^)^ The percent of joints displaying microscopic tissue damage (stained with calcein) or overt fracture of the cortical shell in any of the microscopic sections was determined (Fig. 4*B*). {FIG4} Microscopic tissue damage became apparent at a maximum load magnitude of 6.5 MPa (supraphysiologic load). Maximum load magnitudes greater than 6.5 MPa frequently resulted in fracture of the cortical shell (Fig. 4*C*).

After the preliminary work in cadaver tissue, pilot studies were performed in vivo. Adult male New Zealand white rabbits, 6 to 7 months of age, were prepared for surgery using standard techniques. Animals were intubated and anesthetized through isoflurane inhalation. The right femur was submitted to sham surgery in which the medial surfaces adjacent to the distal condyle were exposed and the aluminum alignment fixture was secured with screws. The fixture was removed after this sham surgery and the surgical wound was closed. Immediately after the sham surgery, the animal was repositioned, and the metallic fixture was placed on the left medial surface. The animal limb was then secured within the materials testing system and the loading platen was slowly inserted through the alignment hole until it contacted the cortex. Cyclic compressive loading was applied from zero to a load sufficient to generate microscopic tissue damage (6.5 MPa, *n* = 2). As an additional control group, two animals were submitted to cyclic loading at a maximum load below the threshold for generation of microscopic tissue damage (5.0 MPa, *n* = 2). At the completion of 10,000 loading cycles, the limb was removed from the materials testing system, the fixture was removed from the limb, the surgical wound was closed, and the animals were allowed to recover from anesthesia and returned to normal cage activity.

Animals underwent MRI with anesthesia at 2, 3, 4, 6, and 9 weeks after surgery, or until a BML was no longer observed. Images acquired included: a 3‐plane proton density‐weighted fast spin echo series, STIR images in the axial and coronal planes, IDEAL (two‐point Dixon technique) images in axial and coronal planes, and a sagittal plane T2 mapping series for quantitative evaluation of articular cartilage. Total image acquisition time was between 30 and 45 minutes. Animals were allowed to recover from anesthesia and returned to cage activity.

Loaded limbs displayed BMLs at the location of applied mechanical loads at 2 weeks after surgery and loading (Fig. 4*C*). Limbs subjected to cyclic loading sufficient to generate microscopic tissue damage in bone (6.5 MPa maximum load) displayed substantially larger BMLs than were observed in limbs experiencing lower‐magnitude loads (5.0 MPa) or sham‐loaded limbs (Fig. 4*C*). Additionally, the volume of BMLs in limbs submitted to the 6.5 MPa maximum load waveform continued to increase until 4 weeks after surgery/loading whereas the BMLs in limbs submitted to 5.0 MPa maximum load decreased in volume and resolved by 4 weeks after surgery/loading (Fig. 4*D*). At 9 weeks after surgery/loading, one BML was still present in a limb loaded with the 6.5 MPa waveform. Limbs submitted to sham surgery displayed a very small volume of BML‐like signal. After bone marrow lesions were no longer observable, animals were euthanized, femurs were dissected from soft tissue, fixed, decalcified, and embedded in paraffin. Coronal sections were collected and stained with hematoxylin and eosin for examination of cartilage adjacent to the location of the BML. Histopathology did not detect signs of cartilage degradation in any of the limbs in this small study, nor did T2 mapping imaging at the end of the study; however, because tissue was collected at only one period after loading, it is unclear if a longer time period after loading is required to observed cartilage damage.

These preliminary studies demonstrate that BMLs can be generated using mechanical loading with only minimal surgical intervention (no overt fracture, no implant) and without directly damaging the overlying cartilage. Additionally, this work demonstrates that BMLs generated in the absence of microscopic tissue damage in underlying cancellous bone are small in volume and resolve readily.

## 7 Conclusions and Outlook

Although there are substantial data supporting a correlation between BMLs and progression of joint and cartilage degeneration, the pathophysiology of BMLs and their potential contribution to osteoarthritis remains an enigma. BMLs remain an enticing target for the development of diagnostics and therapeutics. Animal models provide a means to understand the underlying mechanisms within BMLs by providing a controlled method to examine the natural history of BMLs and associated bone‐cartilage cross‐talk. An appropriate animal model requires substantial model validation that includes detailed characterization of underlying bone's response to mechanical stimulus, especially microscopic tissue damage. Establishing a model requires both mid to large animals (rabbits, dogs, or larger) and frequent MR imaging, so directly addressing these questions will require a substantial commitment from investigators.

## Disclosures

Authors report no conflicts of interest.

8

### Peer Review

The peer review history for this article is available at https://publons.com/publon/10.1002/jbm4.10609.
